# Elucidating the molecular landscape of tendinitis: the role of inflammasome-related genes and immune interactions

**DOI:** 10.3389/fimmu.2024.1393851

**Published:** 2024-06-11

**Authors:** Hongwei Xu, Xiaolang Lu, Yang Yu, Yifei Zhou, Tengfei Qi, Yijing Zheng

**Affiliations:** ^1^ The Second Affiliated Hospital and Yuying Children’s Hospital of Wenzhou Medical University, Wenzhou, China; ^2^ Department of Neurosurgery, Trauma Center, Shandong Provincial Hospital Affiliated to Shandong First Medical University, Jinan, China

**Keywords:** tendinitis, inflammasomes, gene expression, immune system, bioinformatics

## Abstract

Tendinitis, characterized by the inflammation of tendons, poses significant challenges in both diagnosis and treatment due to its multifaceted etiology and complex pathophysiology. This study aimed to dissect the molecular mechanisms underlying tendinitis, with a particular focus on inflammasome-related genes and their interactions with the immune system. Through comprehensive gene expression analysis and bioinformatics approaches, we identified distinct expression profiles of inflammasome genes, such as NLRP6, NLRP1, and MEFV, which showed significant correlations with immune checkpoint molecules, indicating a pivotal role in the inflammatory cascade of tendinitis. Additionally, MYD88 and CD36 were found to be closely associated with HLA family molecules, underscoring their involvement in immune response modulation. Contrary to expectations, chemokines exhibited minimal correlation with inflammasome genes, suggesting an unconventional inflammatory pathway in tendinitis. Transcription factors like SP110 and CREB5 emerged as key regulators of inflammasome genes, providing insight into the transcriptional control mechanisms in tendinitis. Furthermore, potential therapeutic targets were identified through the DGidb database, highlighting drugs that could modulate the activity of inflammasome genes, offering new avenues for targeted tendinitis therapy. Our findings elucidate the complex molecular landscape of tendinitis, emphasizing the significant role of inflammasomes and immune interactions, and pave the way for the development of novel diagnostic and therapeutic strategies.

## 1 Introduction

Tendinitis, characterized by the inflammation of tendons, represents a common musculoskeletal disorder that significantly impacts the quality of life and productivity of affected individuals (1–3). It is a condition that spans a wide demographic, from athletes to the elderly, and from manual laborers to office workers, making it a prevalent concern in both sports medicine and general healthcare (4, 5). The etiology of tendinitis involves a complex interplay of mechanical overuse, age-related degeneration, and inflammatory processes, yet the precise molecular mechanisms underlying its pathogenesis remain insufficiently understood (6, 7).

The inflammatory response in tendinitis is a critical aspect of its pathology. Inflammasomes, as intracellular multiprotein complexes, play a pivotal role in the innate immune system by regulating the activation of inflammatory processes and cytokines such as IL-1β and IL-18 (8, 9). These complexes are involved in detecting pathogenic microorganisms and stress signals, thus initiating an inflammatory response (10). The involvement of inflammasomes in various autoimmune and inflammatory diseases has been extensively documented, yet their specific roles in tendinitis are not fully elucidated (11, 12).

Recent advancements in high-throughput genomic technologies and bioinformatics tools have opened new avenues for exploring the complex biological networks underlying tendinitis (13, 14). Gene expression profiling, coupled with systems biology approaches such as Weighted Gene Co-expression Network Analysis (WGCNA), has proven instrumental in identifying key molecular players and pathways involved in various diseases (15, 16). These methodologies allow for the exploration of gene co-expression networks, identification of disease-associated genes, and elucidation of the molecular mechanisms underlying pathological conditions. Furthermore, the integration of multi-omics data, including transcriptomics and proteomics, with advanced computational analyses, has the potential to reveal novel insights into the pathophysiology of tendinitis (17, 18). Understanding the gene expression patterns and molecular pathways differentially regulated in tendinitis can provide a foundation for identifying novel biomarkers and therapeutic targets (19, 20).

Given the significant burden that tendinitis places on individuals and healthcare systems worldwide, and the gaps in our understanding of its molecular underpinnings, this study aims to dissect the complex molecular landscape of tendinitis. By leveraging high-throughput gene expression data and employing comprehensive bioinformatics analyses, including differential expression analysis, pathway enrichment, and network analysis, we seek to uncover the key molecular pathways and regulatory networks involved in tendinitis. Specifically, we focus on the role of inflammasome genes and their interaction with the immune system, hypothesizing that they play a crucial role in the pathogenesis of tendinitis. The rationale for this study is rooted in the need for a deeper understanding of tendinitis at the molecular level, which is essential for developing more effective diagnostic and therapeutic strategies.

## 2 Materials and methods

### 2.1 Data acquisition

In our comprehensive investigation into the molecular mechanisms underlying tendinitis, we began by sourcing relevant datasets from the Gene Expression Omnibus (GEO), a public repository that aggregates high-throughput gene expression data. We specifically retrieved dataset GSE26051, which consists of gene expression profiles from 23 tendinitis samples and 23 control samples from normal tendons. Additionally, we selected dataset GSE167226 to augment our control group with 19 additional samples of normal tendons. To deepen our analysis, we incorporated a specialized gene set related to inflammasome pathways, curated from the Gene Set Enrichment Analysis (GSEA) official website (21). This set included three distinct pathways known to be associated with inflammasome activity. Through a meticulous process of deduplication, we distilled this information into a list of 26 unique genes directly involved in inflammasome-related functions.

### 2.2 Data preprocessing and batch correction

We combined the datasets from GSE26051 and GSE167226 to increase the robustness of the analysis. The sva package’s ComBat function was employed for batch normalization (22). This process was crucial in mitigating batch effects that are often present when merging datasets from different studies or platforms. We used the boxplot function to visualize the distribution of sample expression levels before and after batch removal. This visualization was instrumental in confirming the efficacy of the batch correction process.

### 2.3 Principal component analysis

Post batch correction, we conducted Principal Component Analysis (PCA) using the FactoMineR and factoextra packages. PCA is a statistical procedure that transforms the data into a set of linearly uncorrelated orthogonal components, providing insight into the underlying structure of the data.

### 2.4 Weighted gene co-expression network analysis

We performed WGCNA using the WGCNA package to construct a gene co-expression network. Setting the soft-thresholding power to 4 allowed us to achieve a scale-free topology fit (R^2 = 0.8), which is indicative of a naturally occurring network. The analysis included the construction of a topological overlap matrix and subsequent module identification. These modules were then correlated with tendinitis to identify genes most associated with the condition.

### 2.5 Expression correlation analysis and gene interaction

Expression correlation analysis was conducted to determine the relationships between core tendinitis genes and the 26 inflammasome-related genes. We used the ggplot2 and dplyr packages to conduct the analysis and generate heatmaps for visualization (23). Additionally, we evaluated the interactions and biological processes associated with these genes through GENEMANIA, an online tool for predicting protein-protein interactions, focusing on Toll-like receptors and the inflammasome assembly processes (24, 25).

### 2.6 Chromosomal distribution

The chromosomal positions of the tendinitis-associated inflammasome genes were depicted using a bar chart, created with the ggplot2 package. This chromosomal mapping provided a genomic context for the inflammasome genes, potentially facilitating further investigations into the genetic basis of tendinitis.

### 2.7 Dataset splitting and machine learning analysis

The consolidated gene dataset was partitioned into training and validation sets utilizing the caret package, with a 70:30 split ratio. This stratification facilitated the development of a robust diagnostic model. We then applied a suite of 12 machine learning algorithms for variable selection and model construction. The algorithms included Lasso, NaiveBayes, Support Vector Machine (SVM), glmBoost, Elastic Net (Enet), Partial Least Squares Regression (plsRglm), Extreme Gradient Boosting (XGBoost), Linear Discriminant Analysis (LDA), Stepwise Generalized Linear Model (Stepglm), Ridge Regression, Random Forest, and Gradient Boosting Machine (GBM).

### 2.8 Model calibration and decision curve analysis

The predictive model, constructed using the 12 inflammasome-related genes, underwent calibration using the rms package to generate calibration curves for both the training and validation sets. This step was critical to assess the model’s accuracy and reliability. Furthermore, we employed the rmda package to perform Decision Curve Analysis (DCA), which quantifies the clinical benefits of the diagnostic model across a range of threshold probabilities.

### 2.9 Differential pathway analysis

To elucidate the disparity in molecular pathways between tendinitis-afflicted and normal tendons, we conducted differential expression analysis using the limma package. Gene Set Enrichment Analysis (GSEA) for Gene Ontology Biological Processes (GO-BP) was performed with the clusterProfiler package, while pathway clustering and visualization were executed using aPEAR.

### 2.10 KEGG pathway enrichment

The 12 inflammasome-related genes were subjected to KEGG pathway enrichment analysis with the clusterProfiler package. The resultant data were visualized using a bubble plot generated by ggplot2, providing a succinct representation of the pathways enriched with the genes of interest.

### 2.11 Immune cell abundance and correlation analysis

The relative abundance of immune cells in tendinitis samples compared to controls was quantified through ssGSEA, performed with the GSVA and GSEABase packages. The graphical representation of immune cell distributions was rendered using ggpubr to create half-violin plots. Subsequent correlation analyses between the 12 inflammasome-related genes and the altered immune cells were conducted using the limma and ggExtra packages, yielding scatter plots that reveal the relationship between gene expression and immune cell changes.

### 2.12 Tumor microenvironment and immune process assessment

To evaluate the tumor microenvironment, we employed the estimate package to calculate scores reflecting the stromal and immune cell compositions of the tendinitis samples. Correlation analyses were then performed using the ggplot2 and dplyr packages to elucidate the relationships between the expression levels of genes and the microenvironmental scores. These scores were instrumental in reflecting the underlying immune and stromal components, which could potentially influence the immunopathogenesis of tendinitis.

Subsequently, we conducted a single-sample Gene Set Enrichment Analysis (ssGSEA) using the GSVA and GSEABase packages to quantify the relative enrichment of different immune processes. To visually represent the intricate associations between the 12 inflammasome-related genes and these immune processes, radar charts were created with the fmsb package, revealing the immunological activity and relevance of these genes.

### 2.13 Immune activity molecule correlation analysis

The relationship between the tendinitis-associated inflammasome genes and various immune activity molecules was explored through a correlation analysis. We utilized the ggplot2 and dplyr packages to construct heatmaps. These visualizations provided a comprehensive overview of the connections between genes and numerous immune checkpoints, as well as the associations between MYD88, CD36, and HLA family molecules. This analysis highlighted the genes’ interactions with immune checkpoints and their potential regulatory roles in immune responses.

### 2.14 Transcription factor prediction and drug analysis

For the prediction of transcription factors regulating the inflammasome-related genes, we accessed the ChEA3 website, which facilitates the identification of transcription factor-gene interactions. The identified top ten transcription factors were then visualized using a bar chart created with ggplot2 to rank them based on their mean rank score.

To elucidate the transcriptional regulatory network, we used Cytoscape software (version 3.9.1) to construct an interaction diagram. This visual tool allowed us to display the complex regulatory relationships between the transcription factors and the inflammasome genes.

Lastly, we explored potential therapeutic agents targeting the inflammasome-related genes by querying the DGIdb database. This database provided a list of drugs with the potential to modulate the activity of these genes. The drug-gene interactions were then depicted in a Sankey diagram, generated using the ggalluvial package, which mapped the predicted therapeutic connections, thereby informing potential tendinitis treatment strategies.

### 2.15 Quantitative real-time PCR

This study was approved by the ethics committee. From December 2023 to January 2024, five patients with tendinitis and five healthy control patients were recruited from the hospital. Peripheral blood mononuclear cells (PBMC) were isolated from the blood samples of these patients using previously described methods. Total RNA was extracted from PBMC samples using the FastPure Cell/Tissue Total RNA Isolation Kit (Vazyme). The extracted RNA was then reverse-transcribed into cDNA using the ReverTra Ace qPCR RT Master Mix and gDNA Remover Kit.

Quantitative real-time PCR (qRT-PCR) was performed using SYBR Premix Ex Taq II in a real-time fluorescence quantitative PCR system, with GAPDH selected as the endogenous control for mRNA. The reaction conditions were as follows: initial denaturation at 95°C for 10 minutes, followed by 45 cycles of 95°C for 5 seconds and 60°C for 30 seconds. Amplifications of the target genes and the internal reference gene were conducted for each sample. Each group of samples included three replicate wells. Data analysis was performed using the 2^(-ΔΔCt) method. Primer sequences are provided in **Supplementary Table 1**.

### 2.16 Statistical analysis

We performed all analyses and visualization in R software (version 4.2.1), unless otherwise stated.

## 3 Result

### 3.1 Data integration and batch correction

**Figure 1:**
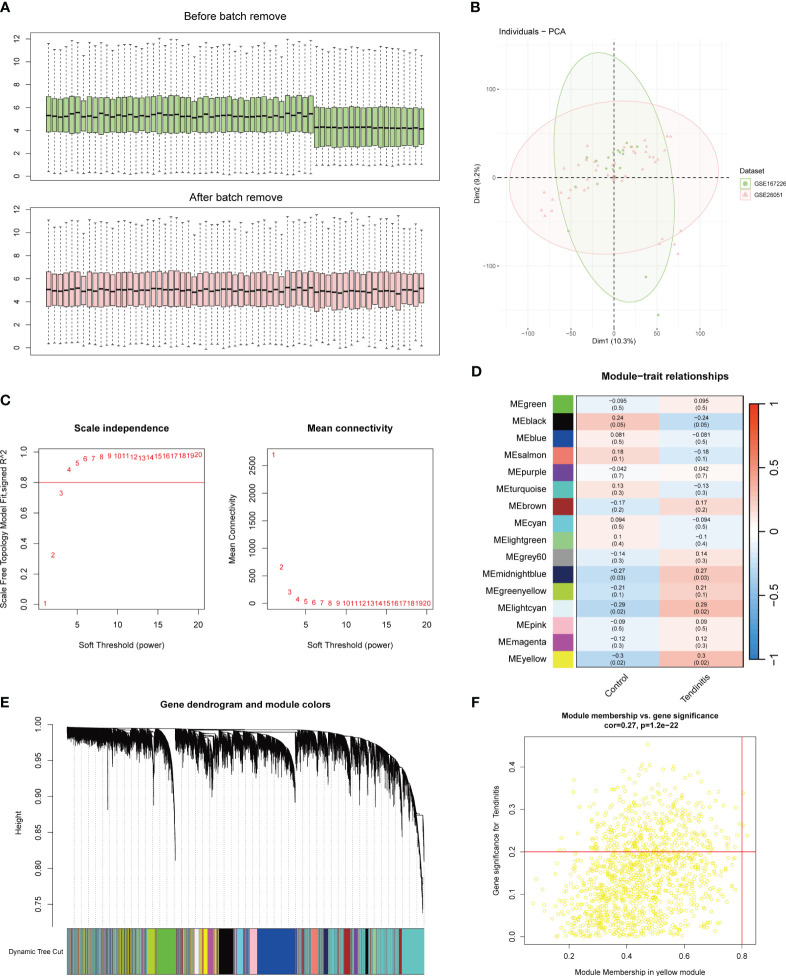
Batch correction and network analysis of tendinitis gene expression. **(A)** Boxplots showing sample average expression levels before and after batch correction. Initially, clear differences are observed between the datasets. Following batch correction using the Combat function of the sva package, expression levels align, demonstrating effective normalization. **(B)** Principal Component Analysis (PCA) of post-batch correction samples. The PCA plot shows a uniform distribution of sample features across datasets, indicating successful batch effect mitigation and suitability for further analysis. **(C)** Analysis of network topology for various soft-thresholding powers in Weighted Gene Co-expression Network Analysis (WGCNA). The left panel shows scale independence as a function of the soft-thresholding power, with a chosen power of 4 achieving the criteria of R^2 = 0.8 for scale-free topology. The right panel displays mean connectivity, affirming the network’s robustness. **(D)** Heatmap of module-trait relationships from WGCNA. Sixteen color-coded gene modules are correlated with tendinitis traits, with the yellow module showing the highest correlation (correlation = 0.3, P < 0.05), suggesting a significant association with tendinitis. **(E)** Dendrogram of genes identified by WGCNA, clustered based on dynamic tree cut, with module colors indicated below. This dendrogram and color band illustrate the gene modules resulting from hierarchical clustering of gene expression data. **(F)** Scatterplot of Module Membership vs. Gene Significance in the yellow module. Genes with a module membership > 0.8 and gene significance > 0.2 are highlighted, identifying ADNP, MSH6, and ZMPSTE24 as key genes associated with tendinitis within this module.

The integration of two datasets was followed by batch correction using the Combat function from the sva package. The boxplot results indicated a clear difference in the average expression levels of samples from the two datasets before batch correction. However, after batch correction, the levels were nearly aligned on the same line (**Figure 1A**). The PCA results post-batch correction showed a uniform mix of sample features, making them suitable for further analysis (**Figure 1B**).

### 3.2 Network analysis with WGCNA

The WGCNA (Weighted Gene Co-expression Network Analysis) results were set with an R^2 value of 0.8. A soft threshold of 4 met the conditions for a scale-free network with good connectivity (**Figure 1C**). Through the calculation of topological matrices and the merging of similar modules, a total of 16 gene modules were identified, each represented by a different color (**Figure 1D**). The correlation matrix revealed that the genes in the yellow module had the highest correlation with tendinitis, with a correlation coefficient of 0.3 and a p-value < 0.05 (**Figure 1E**). Further analysis of the 1269 genes in the yellow module, using a module membership cutoff of 0.8 and gene significance of 0.2, retained only three genes: ADNP, MSH6, and ZMPSTE24, identified as the most tendinitis-associated genes (**Figure 1F**).

### 3.3 Inflammasome gene analysis

**Figure 2:**
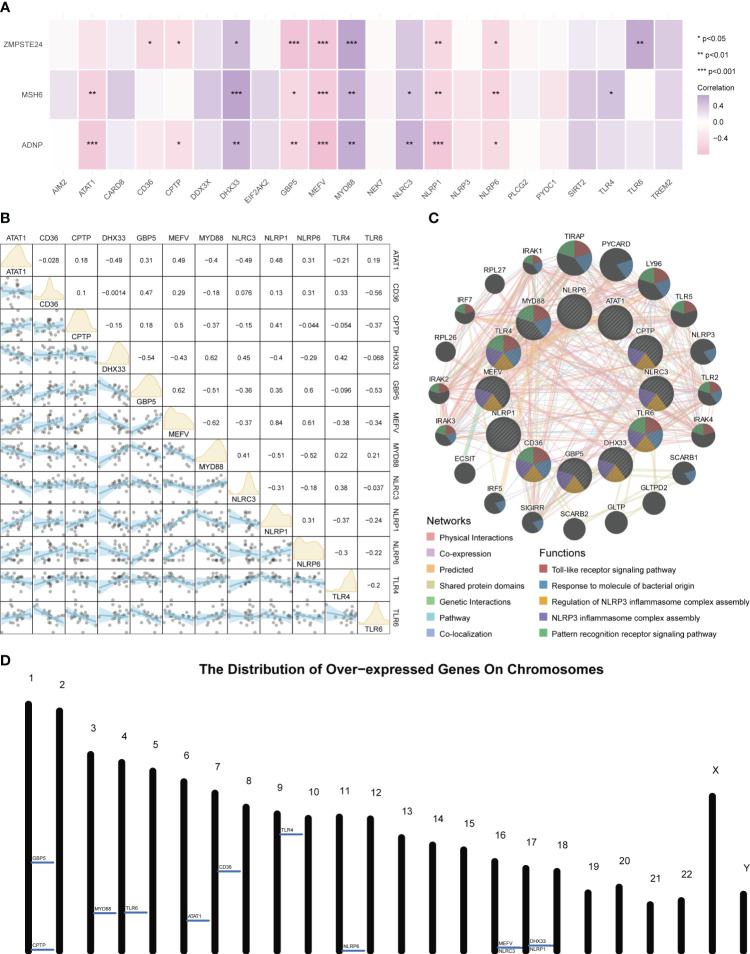
Inflammasome gene correlations and chromosomal localization in tendinitis. **(A)** Correlation heatmap of core tendinitis genes (ADNP, MSH6, ZMPSTE24) and twelve inflammasome-related genes. The heatmap indicates significant positive correlations, suggesting their involvement in inflammasome pathways related to tendinitis. **(B)** Expression correlation matrix for the 12 tendinitis-associated inflammasome genes. The matrix reveals significant correlations between most gene pairs, highlighting potential interactions contributing to the disease mechanism. **(C)** Network visualization from GENEMANIA analysis showing extensive protein-protein interactions among the 12 inflammasome genes, primarily related to Toll-like receptor signaling and inflammasome assembly. **(D)** Chromosomal distribution map of the 12 tendinitis-associated inflammasome genes, illustrating their primary localization on chromosomes 1, 3, 4, 6, 7, 9, 11, 16, and 17, providing insights into their genomic context.

An expression correlation analysis was conducted between these three key tendinitis genes and inflammasome-related genes. The results showed that 12 inflammasome genes were related to the three core tendinitis genes, suggesting these 12 genes are associated with tendinitis-related inflammasomes (**Figure 2A**). The expression correlation analysis among these 12 tendinitis-associated inflammasome genes showed significant and strong correlations for most gene pairs (**Figure 2B**). Analysis using GENEMANIA indicated extensive protein-protein interactions among these genes, primarily involved in biological processes related to Toll-like receptors and inflammasome assembly (**Figure 2C**). A chromosomal bar chart showed that the 12 tendinitis-associated inflammasome genes were mainly located on chromosomes 1, 3, 4, 6, 7, 9, 11, 16, and 17 (**Figure 2D**).

### 3.4 Diagnostic model development

**Figure 3:**
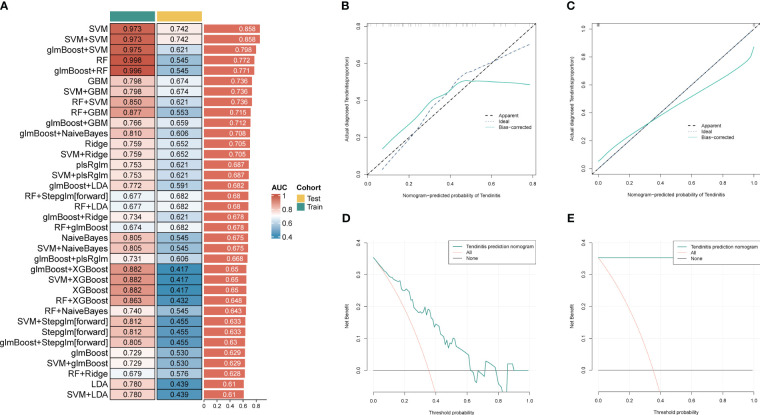
Machine learning models for tendinitis diagnosis based on gene expression. **(A)** Heatmap displaying the performance of 45 machine learning model combinations using various algorithms, including Lasso, NaiveBayes, and SVM. Models are ranked by the average AUC of the ROC curve, with SVM-based models showing the highest diagnostic performance (average AUC = 0.858). **(B, C)** Calibration curves for the predictive model built using the 12 tendinitis-associated inflammasome genes. Curves for both training and validation sets indicate good calibration, suggesting the model’s accuracy in predicting tendinitis. **(D, E)**. Decision Curve Analysis (DCA) for the tendinitis prediction model. The DCA curves demonstrate the model’s clinical usefulness across different threshold probabilities, indicating its potential in improving clinical decision-making.

**Figure 4:**
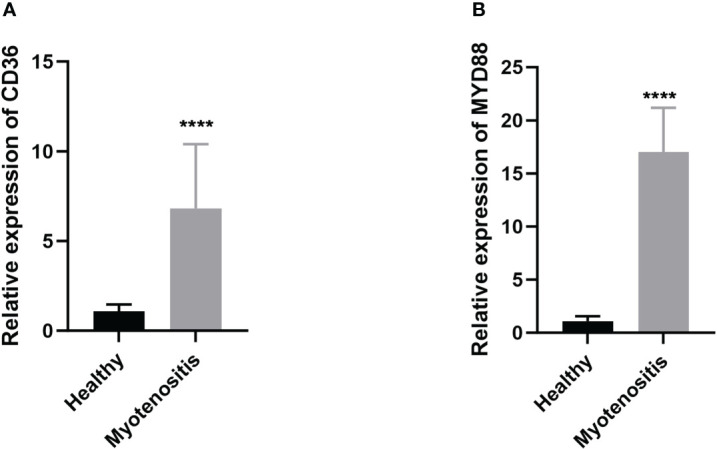
Expression levels of CD36 **(A)** and MYD88 **(B)** in patients with tendinitis.

For further use of these tendinitis-associated inflammasome genes in diagnosing tendinitis, the combined gene set was split into training and validation sets. The caret package was used to divide them into training and validation sets at a ratio of 70% and 30%, respectively. Subsequently, 12 machine learning methods were employed, including Lasso, NaiveBayes, SVM, glmBoost, Enet, plsRglm, XGBoost, LDA, Stepglm, Ridge, RandomForest, and GBM, to perform variable selection and model construction. The variable selection process required a minimum of 2 remaining variables, and only 45 combinations of models were retained. Among these, SVM, used for both gene selection and modeling, achieved the highest diagnostic efficacy with an average AUC of 0.858 under the ROC curve (**Figure 3A**). The predictive model constructed using these 12 tendinitis-associated inflammasome genes demonstrated good calibration in both training and validation sets, as shown by the calibration curves (**Figures 3B, C**). The DCA curves indicated that the diagnostic model could facilitate better clinical decision-making (**Figures 3D, E**). The qPCR results showed that CD36 (**Figure 4A**) and MYD88 (**Figure 4B**) were significantly elevated in patients with tendinitis compared to healthy controls.

### 3.5 Differential pathway analysis in tendinitis

**Figure 5:**
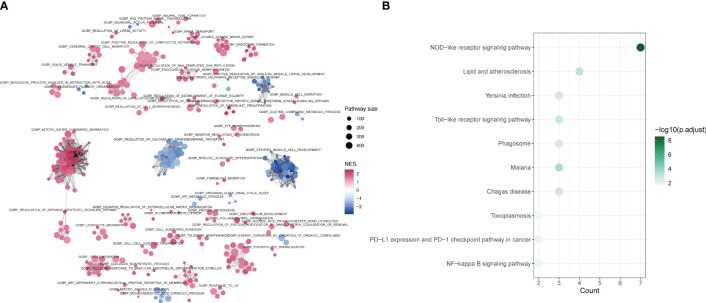
Differential pathway activation in tendinitis via GSEA and KEGG analysis **(A)** Network plot from Gene Set Enrichment Analysis (GSEA) showing clusters of gene sets associated with tendinitis. Gene sets related to intrinsic apoptosis, spindle assembly, and lymphocyte activation are upregulated (red nodes), while pathways involved in calcium ion transport are downregulated (blue nodes), indicating distinct pathway activation profiles in tendinitis. **(B)** Bubble plot of KEGG pathways enrichment analysis for the 12 tendinitis-associated inflammasome genes. Bubble size corresponds to pathway size, and color indicates the normalized enrichment score (NES). Toll-like receptor and Nod-like receptor pathways are significantly enriched, highlighting their potential involvement in tendinitis pathogenesis.

The differences between tendinitis and normal tendons were analyzed through GO-BP GSEA enrichment analysis of differential expression results from both groups. The cluster diagram indicated that tendinitis is primarily associated with the upregulation of processes such as intrinsic apoptosis, spindle assembly, and lymphocyte activation, while calcium-related pathway transport was downregulated (**Figure 5A**). The 12 tendinitis-associated inflammasome genes were mainly enriched in KEGG pathways related to Toll-like receptors and Nod-like receptors (**Figure 5B**).

### 3.6 Immunological aspects in tendinitis

**Figure 6:**
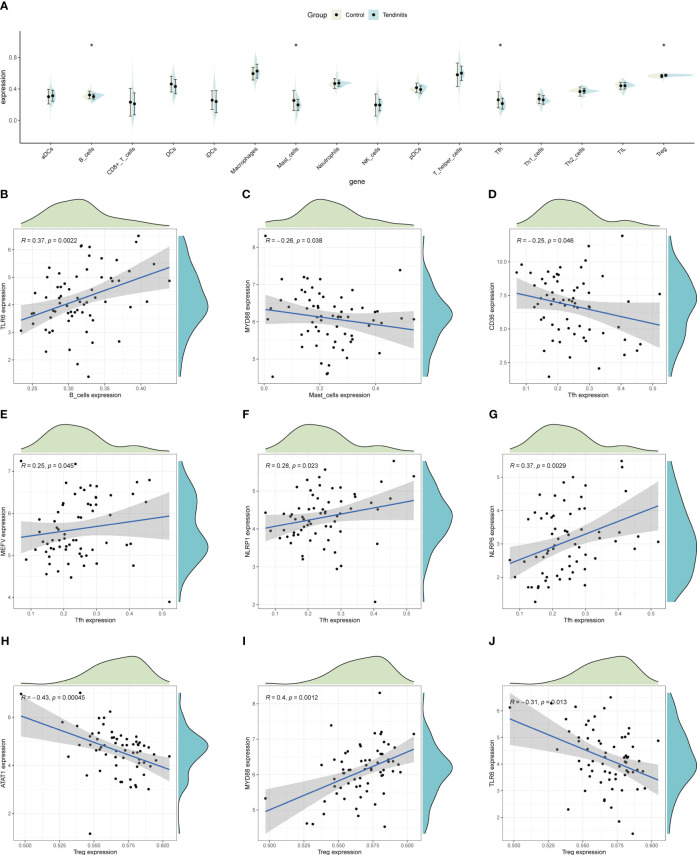
Immune cell abundance and gene correlation in tendinitis. **(A)** Boxplots showing the distribution of immune cell types in tendinitis versus control samples, estimated by ssGSEA. A decrease in B cells, mast cells, and Tfh cells, and an increase in Treg cells in tendinitis samples suggest alterations in the immune cell composition linked to the disease’s immunological environment. **(B)** Positive correlation between B cells and TLR6 expression. **(C)** Negative correlation between mast cells and MYD88 expression. **(D–G)**: Various correlations between Tfh cells and the genes CD36, MEFV, NLRP1, and NLRP6. **(H–J)**: Correlations of Treg cells with ATAT1, TLR6, and MYD88, illustrating complex relationships between inflammasome gene expression and immune cell changes in tendinitis.

ssGSEA was used to estimate the relative abundance of immune cells, revealing that tendinitis samples had fewer B cells, mast cells, and Tfh cells, but more Treg cells compared to control samples, potentially reflecting the immunological environment associated with disease development (**Figure 6A**). To explore whether the 12 tendinitis-associated inflammasome genes were related to changes in these immune cells, a correlation analysis was conducted. B cells showed a positive correlation with the expression of TLR6 (**Figure 6B**), mast cells were negatively correlated with MYD88 (**Figure 6C**), and Tfh cells were negatively correlated with CD36 but positively correlated with MEFV, NLRP1, and NLRP6 (**Figures 6D–G**). Treg cells were negatively correlated with ATAT1 and TLR6 but positively correlated with MYD88 (**Figures 6H–J**).

### 3.7 Tumor microenvironment analysis in tendinitis

**Figure 7:**
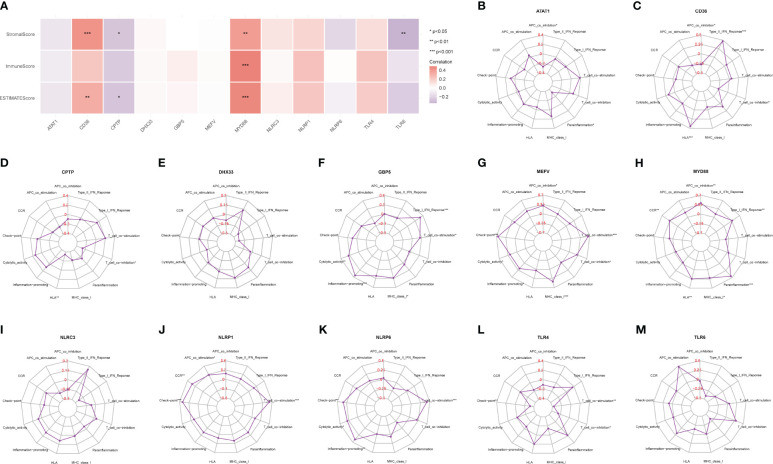
Tendinitis microenvironment and immune process associations. **(A)** Heatmap of the correlation between CD36, MYD88, CPTP, TLR6, and ESTIMATE scores, which assess the immune and stromal components of the tumor microenvironment. The significant positive correlations of CD36 and MYD88 suggest their involvement in promoting an immune microenvironment, while CPTP and TLR6 show significant negative correlations. **(B–M)**: Radar charts depicting the relative abundance of different immune processes and their correlation with the 12 tendinitis-associated inflammasome genes, as assessed by ssGSEA. The charts reveal varying degrees of correlation, with most genes showing significant immunological activity and relevance, except for DHX33, NLRC3, and TLR6, providing a comprehensive view of the immune activity associated with these genes.

An analysis of the tumor microenvironment indicated that CD36 and MYD88 were significantly positively correlated with several microenvironment scores, suggesting their role in promoting the formation of an immune-supportive environment. In contrast, CPTP and TLR6 showed significant negative correlations with these scores (**Figure 7A**). Given the context of tendinitis, it is crucial to understand these correlations as they highlight how certain genes might influence the inflammatory and immune responses within the tendinitis microenvironment. To further elucidate these associations, ssGSEA was used to evaluate the relative abundance of different immune processes and calculate the correlations of 12 tendinitis-associated inflammasome genes with various immune processes, as illustrated in radar charts. The results revealed that, except for DHX33, NLRC3, and TLR6, the remaining genes were correlated to varying degrees with immune processes, indicating their high immunological activity and relevance (**Figures 7B–M**).

### 3.8 Correlation with immune activity molecules

**Figure 8:**
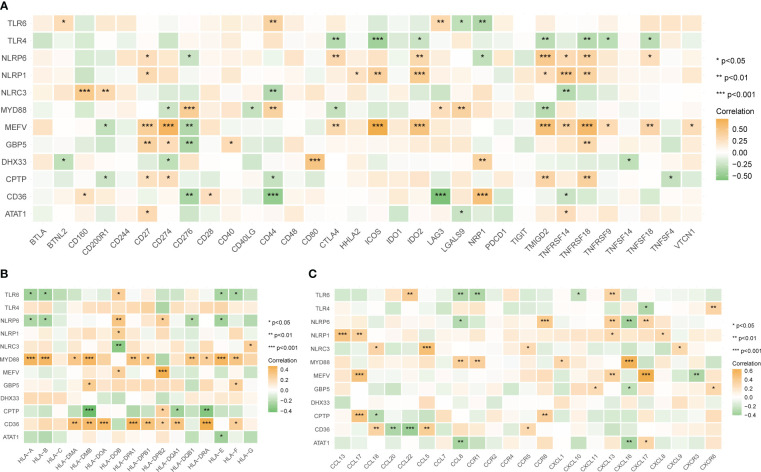
Correlations between tendinitis-associated inflammasome genes and immune molecules. **(A)** Heatmap illustrating the correlation between the 12 tendinitis-associated inflammasome genes and various immune checkpoint molecules. NLRP6, NLRP1, and MEFV exhibit significant positive correlations with numerous immune checkpoints, suggesting their involvement in immune regulation pathways in tendinitis. **(B)** Correlation heatmap showing the relationships between MYD88, CD36, and a spectrum of HLA family molecules. Both MYD88 and CD36 show numerous positive correlations, indicating their potential role in antigen presentation and immune response in tendinitis. **(C)** Heatmap displaying the correlations between chemokines and the 12 tendinitis-associated inflammasome genes. The heatmap suggests minimal expression correlation, indicating a less significant role for these chemokines in inflammasome-related pathways of tendinitis. *P<0.05, **P<0.01, ***P<0.001.

The correlation between the 12 tendinitis-associated inflammasome genes and immune activity molecules was further evaluated. NLRP6, NLRP1, and MEFV showed significant positive correlations with several immune checkpoint molecules (**Figure 8A**). MYD88 and CD36 exhibited the most positive correlations with expressions of HLA family molecules (**Figure 8B**). Chemokines had fewer expression correlations, lacking significant correlations with inflammasome genes (**Figure 8C**).

### 3.9 Regulatory transcription factors of inflammasome genes

**Figure 9:**
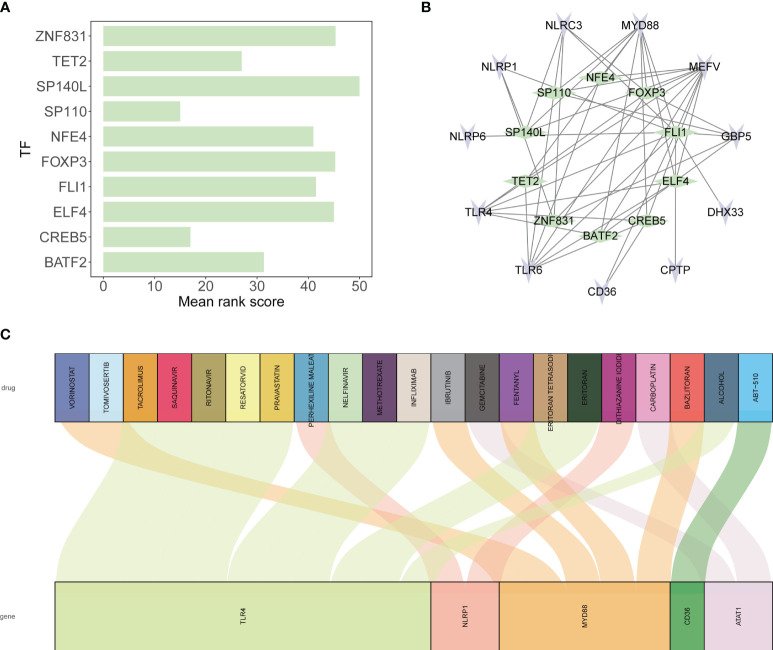
Transcriptional regulation and drug predictions for tendinitis-associated genes. **(A)** Bar graph representing the mean rank scores of the top ten transcription factors (TFs) regulating the 12 tendinitis-associated inflammasome genes. SP110, CREB5, TET2, BATF2, NFE4, FLI1, ELF4, FOXP3, ZNF831, and SP140L are ranked based on their importance in gene regulation, providing insights into the transcriptional control mechanisms in tendinitis. **(B)** Network interaction diagram depicting the regulatory relationships between the identified TFs and the 12 tendinitis-associated inflammasome genes. The diagram highlights the complex interplay between these TFs and genes, suggesting a multifactorial regulatory landscape in tendinitis. **(C)** Sankey diagram derived from the DGidb database, predicting 21 drugs with therapeutic potential targeting the tendinitis-associated inflammasome genes. The diagram links each gene with corresponding drugs, illustrating potential treatment pathways for tendinitis.

The top ten transcription factors regulating the 12 tendinitis-associated inflammasome genes were identified as SP110, CREB5, TET2, BATF2, NFE4, FLI1, ELF4, FOXP3, ZNF831, and SP140L (**Figure 9A**). An interaction diagram displayed the regulatory relationships between these transcription factors and the 12 inflammasome genes (**Figure 9B**). The DGidb database predicted 21 drugs with therapeutic potential against the inflammasome genes, which could be used for the treatment of tendinitis (**Figure 9C**).

## 4 Discussion

The findings of this study shed light on the complex molecular underpinnings of tendinitis, highlighting the significant role of inflammasome-related genes and their interactions with the immune system. The integration and comprehensive analysis of gene expression data have provided valuable insights into the pathophysiological mechanisms of tendinitis, offering potential avenues for novel diagnostic and therapeutic strategies.

Our analysis revealed a distinct expression profile of inflammasome-related genes in tendinitis, with NLRP6, NLRP1, and MEFV showing significant correlations with immune checkpoint molecules. This is consistent with existing literature that underscores the involvement of inflammasomes in various inflammatory and autoimmune conditions. For instance, studies have demonstrated the pivotal role of NLRP3 in gout and pseudogout, highlighting the therapeutic potential of targeting inflammasome pathways in inflammatory diseases (26, 27). The positive correlation between certain inflammasome genes and immune checkpoint molecules observed in our study further emphasizes the intricate link between inflammasome activation and immune regulation in tendinitis.

The analysis of immune cell abundance and gene correlations in tendinitis provides significant insights into the immune dynamics associated with the condition. There is a marked decrease in B cells and T follicular helper (Tfh) cells, which indicates a reduction in T-dependent B cell responses. This suggests that in tendinitis, there is an elevated T-independent B cell response, primarily mediated by Toll-like receptors (TLRs) and MYD88. The predominance of this pathway underscores the critical role of innate immunity in tendinitis, highlighting how the interaction between TLRs and MYD88 facilitates a rapid immune response independent of T cell help. This observation is essential as it points to potential therapeutic targets within the TLR-MYD88 signaling axis, which could be leveraged to modulate immune responses and reduce inflammation in tendinitis. Understanding these mechanisms provides a clearer picture of the immune landscape in tendinitis and suggests new avenues for targeted intervention.

Moreover, the differential expression of HLA family molecules, particularly in relation to MYD88 and CD36, aligns with previous findings on the role of HLA genes in immune response and autoimmune diseases (28, 29). The HLA system’s involvement in antigen presentation and immune response modulation is well-documented, and our results suggest a similar mechanism at play in the context of tendinitis.

The reduced expression of chemokines and their lack of significant correlation with inflammasome genes in our study diverges from the established understanding of chemokines as key mediators of inflammation and immune cell recruitment. This discrepancy may indicate a unique inflammatory landscape in tendinitis, where inflammasome activation does not follow the conventional chemokine-driven pathway, or it may reflect the complex regulatory mechanisms that govern chemokine expression in tendinitis.

The identification of key transcription factors, such as SP110 and CREB5, involved in the regulation of inflammasome genes, provides new insights into the transcriptional control mechanisms in tendinitis. These transcription factors have been implicated in immune response regulation and inflammatory processes in other contexts, suggesting a conserved regulatory network that extends to tendinitis.

Comparing our findings with existing studies, the role of inflammasomes in tendinitis appears to be more complex and multifaceted than previously understood (30, 31). The significant correlations between inflammasome genes and immune cells, particularly Treg cells, highlight the dual role of inflammasomes in both promoting and regulating inflammation. This duality is crucial for maintaining immune homeostasis and preventing uncontrolled inflammation, which is a hallmark of tendinitis.

The therapeutic potential of targeting inflammasome pathways, as suggested by the DGidb database predictions, aligns with emerging trends in drug development for inflammatory diseases. The identification of drugs that can modulate the activity of inflammasome genes offers a promising avenue for the development of targeted therapies for tendinitis. This approach is particularly appealing given the limitations and adverse effects associated with current tendinitis treatments, such as nonsteroidal anti-inflammatory drugs (NSAIDs) and corticosteroids. Additionally, our study highlights key molecular pathways, immune cells, and transcription factors involved in tendinitis, such as the roles of MYD88, CD36, CREB5, and ELF4. By elucidating the complex interactions within the immune microenvironment and the regulatory networks of inflammasome genes, our findings provide valuable insights into potential new drug targets. These molecular insights can facilitate the development of more effective and personalized therapeutic strategies, reducing the reliance on broad-spectrum anti-inflammatory medications and minimizing their side effects. Future research focusing on these identified pathways and regulatory mechanisms could lead to innovative treatments that specifically address the underlying molecular causes of tendinitis, ultimately improving patient outcomes.

Despite the valuable insights provided by this study, several limitations must be acknowledged. Due to the constraints of using public databases, we were unable to obtain detailed clinical information for all patients, such as gender, age matching with controls, and presence of inflammatory symptoms. This information is crucial to determine whether the described results are based on data from patients with inflammatory tendinitis, tendinitis due to mechanical overuse or overload, or age-related degenerative conditions. This aspect is key to discussing the context of the obtained results and answering whether inflammasome pathways are associated with inflammatory or degenerative tendinitis. Additionally, the heterogeneity of the samples might also explain why chemokine-associated genes were less affected, particularly if the samples were primarily from degenerative tendinitis cases. Considering these limitations, our findings should be interpreted with caution, and further studies are needed to validate and expand upon these results.

In conclusion, our study contributes to a deeper understanding of the molecular mechanisms underlying tendinitis, with a particular emphasis on the role of inflammasomes and their interaction with the immune system. The findings highlight the potential for novel diagnostic markers and therapeutic targets, paving the way for more effective and targeted interventions in the management of tendinitis. Future research should focus on validating these potential targets in clinical settings and exploring the mechanistic pathways in greater detail to unravel the complex interplay between inflammasomes, immune cells, and the inflammatory response in tendinitis.

## Data availability statement

The original contributions presented in the study are included in the article/**Supplementary Material**. Further inquiries can be directed to the corresponding author.

## Ethics statement

The studies involving humans were approved by Second Affiliated Hospital and Yuying Children’s Hospital of Wenzhou Medical University. The studies were conducted in accordance with the local legislation and institutional requirements. The participants provided their written informed consent to participate in this study.

## Author contributions

HX: Data curation, Writing – original draft. XL: Data curation, Writing – review & editing. YY: Data curation, Writing – review & editing. YFZ: Data curation, Writing – review & editing. TQ: Writing – review & editing, Conceptualization. YJZ: Conceptualization, Writing – review & editing.
