# Supplementary material for: Elucidating the molecular landscape of tendinitis: the role of inflammasome-related genes and immune interactions
Source: Front Immunol. 2024 Jun 11;15:1393851. doi: 10.3389/fimmu.2024.1393851 (PMC11196777; doi:10.3389/fimmu.2024.1393851)
Supplement: Supplementary file 1 [file Table_1.docx]

**Supplementary table 1. A list of primers used in this study.**

| Gene | Forward sequence (5’ to 3’) | Reverse sequence (5’ to 3’) |
| --- | --- | --- |
| GAPDH | GGAGCGAGATCCCTCCAAAAT | GGCTGTTGTCATACTTCTCATGG |
| MYD88 | GGCTGCTCTCAACATGCGA | CTGTGTCCGCACGTTCAAGA |
| CD36 | GGCTGTGACCGGAACTGTG | AGGTCTCCAACTGGCATTAGAA |
